# Supplementary material for: Caffeine-Containing Energy Shots Cause Acute Impaired Glucoregulation in Adolescents
Source: Nutrients. 2020 Dec 16;12(12):3850. doi: 10.3390/nu12123850 (PMC7766305; doi:10.3390/nu12123850)
Supplement: Supplementary file 1 [file nutrients-12-03850-s001.pdf]

Caffeine-Containing Energy Shots cause Acute Impaired Glucoregulation in Adolescents

Jane Shearer, Raylene A. Reimer, Dustin S. Hittel, Mackenzie A. Gault, Hans J. Vogel, Matthias S. Klein

Supplemental Material

Tables

Table S1. Serum compounds significantly correlating with caffeine intake (DECAF vs. CAF) or caffeine metabolism score (Low vs. High). Given are raw p-values and correlation coefficients from general linear model analysis. *Significant after correction for multiple testing.

Chemical shift [ppm]	Metabolite ID	p-values		Coefficients	
		Slow vs. Fast Caffeine Metabolism Score	DECAF vs. CAF	Slow vs. Fast Caffeine Metabolism Score	DECAF vs. CAF
**8.35,8.33**	folic acid	9.67E-01	6.97E-03*	3.61E+01	9.40E+02
**7.913,7.9**	caffeine	1.33E-01	4.02E-16*	6.07E+02	4.53E+03
**6.915,6.885**	?	1.04E-02*	8.73E-01	-2.75E+03	-1.63E+02
**5.51,5.5**	?	9.30E-03*	3.03E-01	1.57E+03	5.90E+02
**5.25,5.24**	glucose	2.41E-03*	2.47E-02	1.19E+05	8.37E+04
**5.24,5.23**	glucose	3.27E-03*	2.80E-02	1.19E+05	8.49E+04
**5.1935,5.184**	?	1.67E-01	1.59E-02*	1.79E+03	3.07E+03
**4.34,4.33**	tartaric acid	4.99E-01	3.24E-05*	-9.23E+02	-5.50E+03
**4.18,4.17**	?	1.14E-02*	2.74E-01	3.07E+03	1.26E+03
**4.17,4.16**	?	1.70E-02*	1.72E-01	2.61E+03	1.43E+03
**4.16,4.15**	lactic acid	5.43E-02	1.07E-02*	2.05E+03	2.68E+03
**4.14,4.13**	lactic acid	3.66E-01	3.91E-03*	6.43E+03	2.00E+04
**4.13,4.12**	lactic acid	4.52E-02	6.22E-03*	4.12E+04	5.57E+04
**4.12,4.11**	lactic acid	2.42E-02	7.52E-03*	4.68E+04	5.47E+04
**4.11,4.1**	lactic acid	2.09E-02	6.36E-03*	1.82E+04	2.12E+04
**4.02,4.01**	?	1.26E-03*	9.17E-02	5.09E+03	2.49E+03
**3.97,3.965**	caffeine	3.16E-01	2.35E-15*	4.13E+03	4.09E+04
**3.95,3.94**	1,7-dimethylxanthine	1.72E-02*	1.90E-03*	5.11E+03	6.62E+03
**3.93,3.92**	glucose	1.07E-02*	5.49E-02	1.63E+04	1.18E+04
**3.92,3.91**	glucose	2.80E-03*	2.75E-02	1.39E+05	9.76E+04
**3.91,3.9**	glucose	7.38E-03*	8.46E-01	2.52E+04	1.73E+03
**3.9,3.89**	glucose	3.09E-03*	2.97E-02	1.80E+05	1.26E+05
**3.89,3.88**	glucose	1.50E-02*	1.46E-01	8.83E+03	5.06E+03
**3.87,3.86**	glucose	2.09E-03*	2.98E-02	4.42E+04	2.96E+04
**3.85,3.84**	glucose	2.58E-03*	2.48E-02	1.43E+05	1.02E+05
**3.84,3.83**	glucose	2.72E-03*	2.97E-02	1.35E+05	9.34E+04

**3.83,3.82**	glucose	2.05E-03*	2.71E-02	7.47E+04	5.08E+04
**3.82,3.81**	glucose	6.88E-03*	6.86E-02	3.06E+04	1.97E+04
**3.76,3.75**	glucose	5.41E-03*	8.05E-02	3.52E+04	2.10E+04
**3.75,3.74**	glucose	2.40E-03*	2.84E-02	9.89E+04	6.79E+04
**3.74,3.73**	glucose	2.44E-03*	2.22E-02	1.51E+05	1.08E+05
**3.73,3.72**	glucose	2.25E-03*	2.76E-02	1.34E+05	9.21E+04
**3.72,3.71**	glucose	3.80E-03*	3.11E-02	1.50E+05	1.06E+05
**3.71,3.7**	glucose	2.45E-03*	2.95E-02	7.42E+04	5.07E+04
**3.7,3.69**	glucose	3.97E-03*	7.47E-01	1.45E+04	1.53E+03
**3.54,3.53**	glucose	2.63E-03*	3.43E-02	7.73E+04	5.17E+04
**3.53,3.52**	glucose	1.42E-02*	6.33E-02	3.45E+04	2.51E+04
**3.52,3.51**	glucose	8.69E-03*	1.05E-01	5.19E+04	3.06E+04
**3.51,3.5**	glucose	1.42E-02*	4.30E-02	4.01E+04	3.19E+04
**3.5,3.49**	glucose	1.98E-03*	2.30E-02	1.98E+05	1.38E+05
**3.48,3.47**	glucose	4.67E-03*	2.93E-02	1.51E+05	1.11E+05
**3.47,3.46**	glucose	2.80E-03*	2.78E-02	1.37E+05	9.63E+04
**3.46,3.45**	glucose	4.26E-03*	3.09E-02	1.11E+05	8.00E+04
**3.44,3.43**	glucose	7.98E-03*	2.25E-02	5.66E+04	4.70E+04
**3.43,3.42**	glucose	3.01E-03*	2.70E-02	1.82E+05	1.29E+05
**3.42,3.41**	glucose	2.31E-03*	2.05E-02	1.39E+05	1.01E+05
**3.41,3.4**	glucose	3.61E-03*	2.36E-02	1.86E+05	1.38E+05
**3.4,3.39**	glucose	3.39E-03*	2.87E-02	6.50E+04	4.63E+04
**3.39,3.38**	glucose	5.99E-03*	2.48E-02	8.88E+03	6.98E+03
**3.38,3.37**	glucose	9.99E-04*	6.61E-02	5.94E+03	3.10E+03
**3.335,3.327**	1,7Dimethylxanthine	2.16E-01	2.09E-03*	-1.87E+03	5.25E+03
**3.27,3.26**	glucose	1.14E-03*	7.97E-01	1.36E+05	9.97E+03
**3.26,3.25**	glucose	3.66E-03*	4.26E-02	9.18E+04	6.10E+04
**3.25,3.24**	glucose	4.46E-03*	2.74E-02	1.34E+05	9.92E+04
**3.24,3.23**	glucose	2.12E-03*	3.68E-02	9.64E+04	6.20E+04
**3.21,3.2**	choline	8.77E-03*	3.19E-04*	6.82E+03	-1.01E+04
**3.16,3.15**	?	9.60E-03*	6.27E-01	7.09E+03	-1.27E+03
**2.73,2.72**	?	9.15E-03*	6.01E-01	1.09E+03	-2.09E+02
**2.7,2.68**	citric acid	7.82E-03*	6.20E-02	4.49E+03	3.01E+03
**2.675,2.655**	citric acid	5.75E-03*	5.65E-02	6.42E+03	4.23E+03
**2.57,2.56**	?	3.02E-01	3.53E-03*	4.01E+02	1.11E+03
**2.56,2.54**	citric acid	2.25E-03*	3.06E-02	7.03E+03	4.72E+03
**2.54,2.534**	citric acid	4.29E-03*	2.25E-01	1.22E+03	4.88E+02
**2.534,2.515**	citric acid	3.03E-03*	2.33E-02	5.38E+03	3.93E+03
**2.37,2.36**	proline	4.52E-03*	8.73E-01	-3.19E+03	1.69E+02
**2.36,2.35**	proline	3.22E-03*	7.14E-01	-4.36E+03	5.11E+02
**2.35,2.34**	proline	7.13E-04*	2.53E-01	-4.86E+03	1.53E+03
**2.34,2.33**	?	1.02E-02*	1.35E-01	-3.64E+03	2.08E+03
**2.33,2.32**	folic acid	2.55E-01	6.12E-03*	-7.32E+02	1.96E+03
**2.3,2.29**	?	1.93E-01	9.64E-03*	-7.10E+02	1.54E+03
**2.07,2.06**	proline	2.52E-03*	7.65E-01	-3.65E+03	-3.36E+02
**2.06,2.05**	proline	2.15E-03*	8.83E-01	-3.85E+03	-1.73E+02
**2.04,2.03**	proline	4.71E-03*	5.67E-01	-3.06E+03	5.88E+02
**2.03,2.02**	proline	3.45E-03*	7.12E-01	-6.10E+03	7.25E+02
**2.02,2.01**	proline	6.59E-03*	6.81E-01	-8.92E+03	1.28E+03
**2.01,2**	proline	8.71E-03*	5.91E-01	-8.43E+03	1.65E+03
**2,1.99**	proline	3.56E-03*	8.62E-01	-5.34E+03	2.99E+02
**1.72,1.71**	?	1.66E-02*	2.86E-01	-3.47E+03	-1.48E+03
**1.71,1.7**	?	1.11E-02*	2.44E-01	-2.56E+03	-1.12E+03
**1.36,1.35**	?	1.68E-02*	1.84E-01	3.06E+03	1.63E+03
**1.34,1.33**	lactic acid	3.77E-02	8.44E-03*	2.03E+05	2.53E+05
**1.33,1.32**	lactic acid	3.72E-02	9.87E-03*	2.09E+05	2.55E+05
**1.31,1.3**	lactic acid	3.15E-01	1.12E-02*	1.01E+03	2.50E+03

**1.13,1.12**	?	2.97E-02	1.46E-02*	1.32E+03	1.46E+03
**1.03,1.02**	?	1.72E-02*	9.55E-01	-1.20E+03	2.75E+01
**0.98,0.97**	?	1.64E-02*	4.83E-01	-6.36E+03	-1.77E+03
**0.92,0.91**	?	1.45E-02*	9.05E-02	4.04E+03	2.69E+03
**0.91,0.9**	?	2.11E-03*	7.51E-02	8.82E+03	4.80E+03
**0.9,0.89**	?	1.01E-02*	1.77E-01	3.61E+03	1.80E+03

Figures

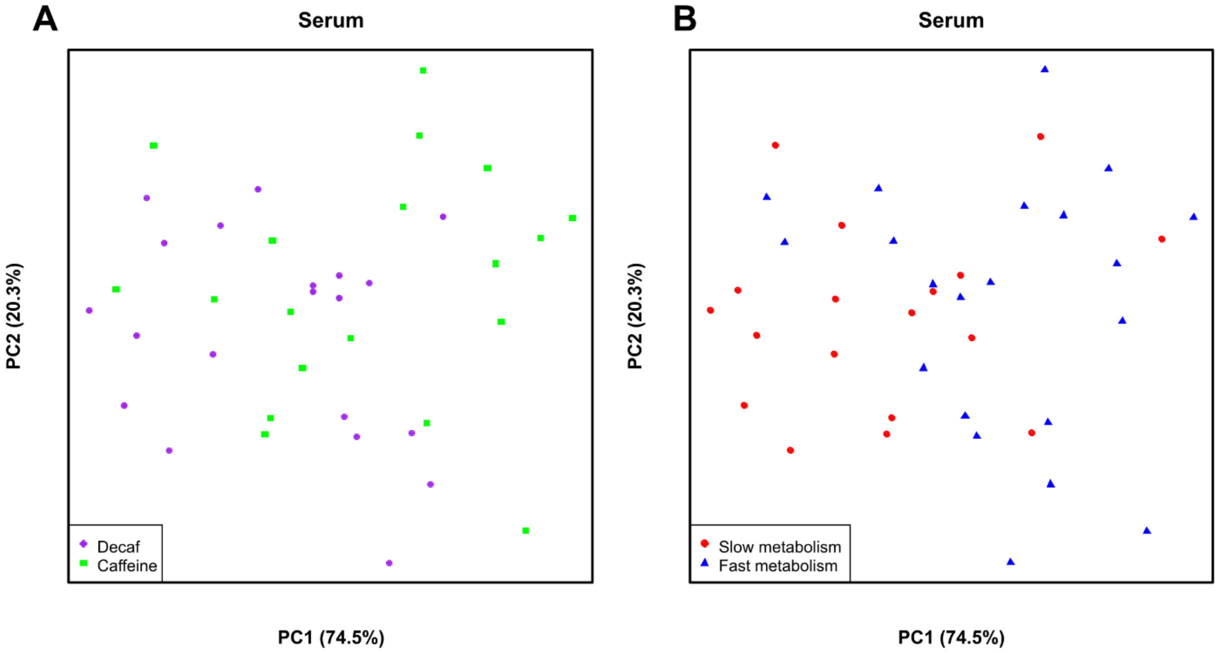

Figure S1. Serum metabolomics (1H-NMR) of 120min time point of each OGTT. (A) PCA analysis of the full NMR data set, showing DECAF and CAF treatments. (B) PCA analysis showing slow and fast allele scores.
